# A Comparative Study of Triclosan-Coated Suture Versus Conventional Suture on Surgical Site Infections of Abdominal Fascial Closures in Open Appendectomy

**DOI:** 10.7759/cureus.85392

**Published:** 2025-06-05

**Authors:** Shreeya Doddannavar, Manjunath Kotennavar, Aravind Patil, Manjunath S Savant, Pradeep P Jaju, Sanjeev Rathod, Veena Ghanteppagol, Narendra Ballal, Eswar Medikonda, Divyang GB

**Affiliations:** 1 General Surgery, Shri B. M. Patil Medical College, Hospital and Research Centre, BLDE (Deemed to be University), Vijayapura, IND; 2 Surgery, Shri B. M. Patil Medical College, Hospital and Research Centre, BLDE (Deemed to be University), Vijayapura, IND; 3 General Surgery, Kasturba Medical College, Manipal, Udupi, IND

**Keywords:** open appendectomy, pds suture, pus culture and sensitivity, ssi, surgical site infection, tcs triclosan coated suture, wound dehiscence

## Abstract

Background

Surgical site infections (SSIs) remain a significant challenge following appendectomy procedures, contributing to increased morbidity, prolonged hospitalization, and substantial healthcare costs. This study aimed to compare the efficacy of triclosan-coated polydioxanone sutures (PDS Plus Antibacterial (polydioxanone) Suture; Ethicon Inc., Raritan, New Jersey, United States) with conventional polydioxanone sutures (PDS II (polydioxanone) Suture; Ethicon Inc.) in preventing SSIs following abdominal fascial closure in open appendectomy.

Methods

This prospective comparative study was conducted at a tertiary care hospital in India between March 2023 and January 2025. A total of 114 patients undergoing open appendectomy were equally allocated to receive either conventional PDS II suture (n=57) or triclosan-coated PDS Plus suture (n=57) for abdominal fascial closure. The primary outcome was the incidence of SSI, with secondary outcomes including type of SSI, microbiological profile, hospital stay duration, and associated factors.

Results

Demographics and clinical characteristics were comparable between groups. SSI occurred in significantly fewer patients in the PDS Plus group compared to the PDS II group (1.8% vs. 19.3%, p=0.002). Deep SSIs were observed exclusively in the PDS II group (8.8%), with none in the PDS Plus group (p=0.008). Wound cultures revealed fewer pathogenic bacteria isolates in the PDS Plus group (p=0.04). The mean length of hospital stay was significantly shorter with triclosan-coated sutures (3.58±1.17 vs. 5.51±1.6 days, p<0.001). Patients who developed SSI, regardless of suture type, had significantly longer hospitalizations (7.75±1.7 vs. 4.17±1.2 days, p<0.001). No significant associations were found between comorbidities or diagnostic categories and SSI occurrence.

Conclusion

Triclosan-coated polydioxanone sutures significantly reduce SSI rates and hospital stay duration in open appendectomy procedures. The complete absence of deep SSIs and substantially fewer pathogenic bacteria isolates in the triclosan-coated suture group suggests a potent protective effect against serious infections. These benefits likely offset the higher unit cost of antimicrobial sutures, supporting their use in open appendectomy and potentially other procedures with substantial infection risk.

## Introduction

Surgical site infections (SSIs) remain one of the most common and challenging complications following both open and laparoscopic surgical procedures, representing a significant burden to healthcare systems worldwide [1]. Despite advances in surgical techniques, perioperative care, and antimicrobial prophylaxis, SSIs continue to affect approximately 2-5% of patients undergoing clean extra-abdominal operations and up to 20% of patients undergoing intra-abdominal procedures [2]. These infections are associated with prolonged hospitalization, increased healthcare costs, additional surgical interventions, and in some cases, life-threatening complications leading to significant morbidity and mortality. The economic impact of SSIs is substantial, with studies estimating that each SSI adds an average of 7-10 additional hospital days and approximately $20,000-$30,000 in extra costs per patient [3].

Appendectomy, the surgical removal of the appendix, is one of the most commonly performed emergency surgical procedures worldwide, with reported incidence rates of SSIs ranging from 1-10% in laparoscopic procedures to 3-15% in open appendectomy, particularly in cases of complicated appendicitis [4]. The pathogenesis of SSIs is multifactorial and involves complex interactions between patient-related factors, surgical techniques, and microbial characteristics [5]. Among the various aspects of surgical technique, the method of wound closure and the choice of suture material have been recognized as modifiable factors potentially influencing SSI rates. Surgical sutures can paradoxically serve as a nidus for bacterial attachment and colonization, potentially contributing to wound infection [6]. This phenomenon is particularly relevant in contaminated or potentially contaminated procedures such as appendectomy.

Triclosan (2,4,4'-trichloro-2'-hydroxydiphenyl ether) acts by inhibiting bacterial fatty acid synthesis, thereby disrupting cell membrane integrity and leading to bacterial cell death. This mechanism provides activity against common surgical pathogens frequently implicated in SSIs following abdominal procedures [7]. Triclosan-coated sutures were first introduced in 2002, and their theoretical benefits have been investigated in numerous studies, demonstrating reduced bacterial adherence and colonization compared to conventional non-coated sutures [8]. However, the translation of these promising laboratory findings into clinical practice has yielded inconsistent results. While the World Health Organization (WHO) included a conditional recommendation for the use of triclosan-coated sutures in its 2018 guidelines for the prevention of SSIs [9], the efficacy of these sutures in specific surgical procedures, including appendectomy, remains a subject of ongoing investigation, particularly regarding their effect on fascial closure infections and cost-effectiveness considerations [10,11].

This study aimed to determine the clinical efficacy of triclosan-coated sutures compared to conventional sutures in reducing the incidence and complications of postoperative SSIs. The primary objective was to assess and compare the incidence of SSI between the case group (triclosan-coated suture) and the control group (conventional suture). Secondary objectives included comparing key clinical outcomes such as postoperative pain, associated complications, and duration of hospital stay.

## Materials and methods

This prospective comparative study was conducted in the Department of General Surgery at Shri B.M. Patil Medical College Hospital and Research Centre, B.L.D.E. (Deemed to be University), Vijayapura, India, from March 2023 to January 2025. Ethical clearance for the study was obtained from the Institutional Ethics Committee of B.L.D.E. (Deemed to be University) (registration number: BLDE(DU)/IEC/923/2023-24, dated April 10, 2023).

Eligibility criteria

The study included patients aged 18-60 years who presented to the outpatient department or emergency services with appendicitis and subsequently underwent open appendectomy. Patients with compromised immune systems and pregnant women with acute appendicitis were excluded from the study.

Study population

The study population was divided into two groups: Group 1 patients underwent abdominal fascial closure using conventional polydioxanone sutures (PDS II (polydioxanone) Suture; Ethicon Inc., Raritan, New Jersey, United States), while Group 2 patients underwent closure using triclosan-coated polydioxanone sutures (PDS Plus Antibacterial (polydioxanone) Suture; Ethicon Inc.). Allocation into groups was carried out in an alternate fashion to ensure even distribution. All patients included in the study underwent either elective or emergency open appendectomy. All layers of the abdominal wall, including peritoneum, muscle, and fascia, were closed using the allocated suture material (either PDS II or triclosan-coated PDS Plus) as per group allocation. Skin closure was done using non-absorbable sutures.

Sample size calculation

The sample size was calculated using G*Power software version 3.1.9.4 (Heinrich Heine University, Düsseldorf, Germany) based on previous studies [12], which reported a surgical site infection rate of 10.7% in the triclosan group and 33.1% in the control group. To achieve a power of 85% for detecting a statistically significant difference between the groups at a 5% significance level, a minimum of 114 patients were required (57 in each group), assuming equal group sizes and using a two-sided exact test for proportions.

Data analysis

Data were entered into Microsoft Excel (Microsoft Corporation, Redmond, Washington, United States) and analyzed using IBM SPSS Statistics for Windows, Version 20 (Released 2011; IBM Corp., Armonk, New York, United States). Continuous variables were summarized as means and standard deviations (SDs), while categorical variables were presented as frequencies and percentages. An independent samples t-test was used to compare normally distributed continuous variables between the two groups. Categorical variables were analyzed using the Chi-square test. A p-value of less than 0.05 was considered statistically significant. All statistical analyses were conducted using two-tailed tests.

## Results

A total of 114 patients were included in the study and divided equally between the conventional PDS II suture group (n=57) and the triclosan-coated PDS Plus group (n=57). The demographic characteristics were comparable between the groups. The most common age group was 21-40 years, comprising 27 (47.4%) patients in the PDS II group and 31 (54.4%) in the PDS Plus group. The distribution of age was not significantly different (p = 0.8972). Male patients predominated in both groups, accounting for 39 (68.4%) in the PDS II group and 42 (73.7%) in the PDS Plus group (p = 0.5355). Regarding clinical diagnoses, acute appendicitis was the most frequent condition in both groups: 21 (36.8%) in the PDS II group and 20 (35.1%) in the PDS Plus group. Other diagnoses, such as recurrent appendicitis, chronic appendicitis, appendicular mass, subacute appendicitis, and perforated appendicitis, were distributed without statistically significant differences between groups (p = 0.8173) (Table 1).

**Table 1:** Demographic and clinical characteristics of study participants A chi-square test was applied and a p-value of less than 0.05 was considered significant.

Characteristics		PDS II (n=57), n (%)	PDS Plus (n=57), n(%)	Chi-square value	p-value
Age group (years)	18-20	11 (19.3%)	10 (17.5%)	0.5962	0.8972
	21-40	27 (47.4%)	31 (54.4%)		
	41-60	18 (31.6%)	15 (26.3%)		
	61-80	1 (1.8%)	1 (1.8%)		
Sex	Female	18 (31.6%)	15 (26.3%)	0.3838	0.5355
	Male	39 (68.4%)	42 (73.7%)		
Diagnosis	Acute appendicitis	21 (36.8%)	20 (35.1%)	2.2242	0.8173
	Appendicular mass	5 (8.8%)	7 (12.3%)		
	Chronic appendicitis	5 (8.8%)	9 (15.8%)		
	Perforated appendicitis	6 (10.5%)	6 (10.5%)		
	Recurrent appendicitis	11 (19.3%)	8 (14%)		
	Subacute appendicitis	9 (15.8%)	7 (12.3%)		

The occurrence of SSI was significantly higher in the PDS II group, with 11 (19.3%) patients affected, compared to only one (1.8%) in the PDS Plus group (p = 0.0022). Among these, deep SSI was noted in five (8.8%) patients in the PDS II group and none in the PDS Plus group, while superficial SSI occurred in six (10.5%) and one (1.8%) patients, respectively (p = 0.0084). Wound cultures in the PDS II group revealed growth of *Proteus vulgaris* in three (5.3%), *Escherichia coli* in two (3.5%), *Klebsiella pneumoniae* in four (7.0%), and *Acinetobacter/Pseudomonas* in two (3.5%) patients. In contrast, only one (1.8%) patient in the PDS Plus group had growth of *Acinetobacter/Pseudomonas*. The proportion of patients showing no microbial growth was significantly higher in the PDS Plus group (n=56, 98.2%) vs. 46 (80.7%) in the PDS II group (p = 0.0354) (Table 2).

**Table 2:** Surgical site infection outcomes between groups A chi-square test was applied and a p-value of less than 0.05 was considered significant. SSI: surgical site infection

Parameters		PDS II (n=57), n (%)	PDS Plus (n=57), n (%)	Chi-square value	p-value
Occurrence of SSI	Yes	11 (19.3%)	1 (1.8%)	9.3137	0.0022
	No	46 (80.7%)	56 (98.2%)		
Type of SSI	Deep	5 (8.8%)	0 (0%)	9.5518	0.0084
	Superficial	6 (10.5%)	1 (1.8%)		
	None	46 (80.7%)	56 (98.2%)		
Wound Culture Results	Proteus vulgaris	3 (5.3%)	0 (0%)	10.3137	0.0354
	Escherichia coli	2 (3.5%)	0 (0%)		
	Klebsiella pneumoniae	4 (7%)	0 (0%)		
	Acinetobacter/Pseudomonas	2 (3.5%)	1 (1.8%)		
	No growth	46 (80.7%)	56 (98.2%)		

Comorbidities were comparable between groups. Diabetes mellitus was present in one (1.8%) patient in each group. Hypertension was noted in six (10.5%) patients in the PDS II group and three (5.3%) in the PDS Plus group. Obesity was highly prevalent, affecting 48 (84.2%) in the PDS II group and 49 (86.0%) in the PDS Plus group. A small number of patients had no comorbidities: two (3.5%) in the PDS II group and four (7.0%) in the PDS Plus group. These differences were not statistically significant (p = 0.6421) (Table 3).

**Table 3:** Comorbidities A chi-square test was applied and a p-value of less than 0.05 was considered significant.

Comorbidities	PDS II (n=57), n (%)	PDS Plus (n=57), n (%)	Statistical value	p-value
Diabetes mellitus	1 (1.8%)	1 (1.8%)	1.6769	0.6421
Hypertension	6 (10.5%)	3 (5.3%)		
Obesity	48 (84.2%)	49 (86%)		
None	2 (3.5%)	4 (7%)		

The mean length of hospital stay was significantly shorter in the PDS Plus group, at 3.58 ± 1.17 days, compared to 5.51 ± 1.6 days in the PDS II group. This difference was statistically significant with a t-value of 7.36 and a p-value < 0.001, indicating better postoperative recovery with triclosan-coated sutures (Table 4).

**Table 4:** Length of hospital stay A t-test was applied and a p-value of less than 0.05 was considered significant.

Length of hospital stay (days)	PDS II (n=57)	PDS Plus (n=57)
Mean ± SD	5.51 ± 1.6	3.58 ± 1.17
t-value	7.36	
p-value	<0.001	

A total of 38 patients underwent elective open appendectomy, while 76 underwent emergency procedures. The incidence of SSI was higher among patients operated in emergency settings (n=11/76, 14.5%) compared to those operated selectively (n=1/38, 2.6%), but this difference did not reach statistical significance (χ² = 3.175, p = 0.0747).

An analysis of factors associated with SSI revealed no statistically significant differences in comorbidities between patients with and without infection. Among patients with SSI (n = 12), 10 (83.3%) were obese, and one (8.3%) had hypertension. Diagnoses among SSI patients included acute appendicitis in five (41.7%), subacute appendicitis in three (25.0%), and one case each of chronic, recurrent, perforated, and appendicular mass appendicitis. These distributions showed no significant associations with SSI (p = 0.8321). However, the mean hospital stay was significantly longer among patients with SSI at 7.75 ± 1.7 days compared to 4.17 ± 1.2 days in those without infection (t = 7.09; p < 0.001) (Table 5).

**Table 5:** Association of SSI with clinical parameters Frequency and percentages were calculated for categorical variables, whereas mean and SD were calculated for continuous variables. A chi-square test and t-test were applied, respectively, and a p-value of less than 0.05 was considered significant. SSI: surgical site infection

Parameters		SSI Absent (n=102)	SSI Present (n=12)	Statistical value	p-value
Comorbidities	Diabetes mellitus	2 (2.0%)	0 (0%)	χ² = 0.4836	0.9224
	Hypertension	8 (7.8%)	1 (8.3%)		
	Obesity	87 (85.3%)	10 (83.3%)		
	None	5 (4.9%)	1 (8.3%)		
Diagnosis	Acute appendicitis	36 (35.3%)	5 (41.7%)	χ² = 2.1216	0.8321
	Appendicular mass	11 (10.8%)	1 (8.3%)		
	Chronic appendicitis	13 (12.7%)	1 (8.3%)		
	Perforated appendicitis	11 (10.8%)	1 (8.3%)		
	Recurrent appendicitis	18 (17.6%)	1 (8.3%)		
	Subacute appendicitis	13 (12.7%)	3 (25%)		
Length of hospital stay (days)	Mean ± SD	4.17 ± 1.2	7.75 ± 1.7	t = 7.09	<0.001

## Discussion

SSIs remain a significant postoperative complication, especially in abdominal procedures like open appendectomy, which are prone to contamination. The use of antimicrobial-coated sutures, particularly triclosan-coated sutures, has been explored as a strategy to reduce SSIs by inhibiting early bacterial colonization at the incision site. In our study, the use of triclosan-coated polydioxanone sutures (PDS Plus) resulted in a significantly lower incidence of SSIs compared to conventional polydioxanone sutures (1(1.8%) vs. 11(19.3%), p=0.002), indicating a substantial protective effect in a high-risk surgical population.

Our findings are in line with previous research demonstrating the efficacy of triclosan-coated sutures. Studies by Nakamura et al. [13], Ruiz-Tovar et al. [14], and meta-analyses by de Jonge et al. [15] have all shown significant reductions in SSI rates across various surgical disciplines. Importantly, in our study, deep SSIs were reported only in the conventional suture group, suggesting that triclosan-coated sutures may provide more robust protection against severe infections. These results highlight the potential role of suture selection in improving surgical outcomes.

Microbial analysis revealed a predominance of gram-negative organisms such as *K. pneumoniae*, *P. vulgaris*, and *E. coli*, particularly in the conventional group. These findings differ slightly from global trends, where gram-positive organisms, especially *Staphylococcus aureus*, are more commonly implicated in SSIs. This variation may reflect local microbial profiles and regional antimicrobial resistance patterns. Notably, triclosan-coated sutures were associated with a marked reduction in infections by these organisms, consistent with their broad-spectrum antimicrobial activity.

We also observed a significantly shorter duration of hospital stay in the triclosan group (mean 3.58 days vs. 5.51 days, p<0.001), underscoring the clinical and economic benefits of reducing postoperative infections. Similar trends have been reported by Thimour-Bergström et al. [16] and Singh et al. [17], who linked antimicrobial sutures to decreased hospital costs and faster recovery. Although comorbidities such as obesity and hypertension are traditionally associated with higher SSI risk, we did not find a statistically significant association in our cohort, possibly due to high baseline prevalence and uniform surgical techniques.

Limitations

This study is not without limitations. The sample size, while adequate for detecting differences in SSI rates, may be insufficient to assess the impact of individual comorbidities or rare complications. Additionally, this was a single-center study, which may limit generalizability to broader populations. Microbial resistance patterns were not longitudinally followed, and cost-effectiveness analysis was not formally conducted. Nevertheless, the results strongly support the incorporation of triclosan-coated sutures as a practical measure to reduce SSIs and associated morbidity in open appendectomies.

## Conclusions

This study demonstrates that triclosan-coated polydioxanone sutures significantly reduce SSIs following open appendectomy. SSI rates were significantly lower with triclosan-coated sutures compared to conventional polydioxanone sutures, with no deep infections occurring in the group using the antimicrobial sutures. Additionally, patients with triclosan-coated sutures had a shorter hospital stay, improving patient recovery and reducing healthcare costs. While cost-effectiveness was not directly assessed, the benefits suggest potential overall savings. The results support routine use of antimicrobial sutures in high-risk procedures like open appendectomy. Further research is needed to confirm these findings in different settings and assess long-term impacts.
